# Oncological transformation in vitro of hepatic progenitor cell lines isolated from adult mice

**DOI:** 10.1038/s41598-022-06427-w

**Published:** 2022-02-24

**Authors:** Rocío Olivera-Salazar, Mariano García-Arranz, Aránzazu Sánchez, Susana Olmedillas-López, Luz Vega-Clemente, Luis Javier Serrano, Blanca Herrera, Damián García-Olmo

**Affiliations:** 1 New Therapies Laboratory, Health Research Institute–Fundación Jiménez Díaz University Hospital (IIS–FJD), Avda. Reyes Católicos, 2, 28040 Madrid, Spain; 2 Department of Surgery, School of Medicine, Universidad Autónoma de Madrid (UAM), Arzobispo Morcillo, 4, 28029 Madrid, Spain; 3 Department of Biochemistry and Molecular Biology, School of Pharmacy, Universidad Complutense de Madrid (UCM), Plaza de Ramón y Cajal, s/n, 28040 Madrid, Spain; 4 Department of Surgery, Fundación Jiménez Díaz University Hospital (FJD), Avda. Reyes Católicos, 2, 28040 Madrid, Spain

**Keywords:** Cancer, Cell biology, Molecular biology, Stem cells, Biomarkers

## Abstract

Colorectal cancer cells can transfer the oncogene *KRAS* to distant cells, predisposing them to malignant transformation (Genometastasis Theory). This process could contribute to liver metastasis; besides, hepatic progenitor cells (HPCs) have been found to be involved in liver malignant neoplasms. The objective of this study is to determine if mouse HPCs—Oval cells (OCs)—are susceptible to incorporate *Kras* GAT (G12D) mutation from mouse colorectal cancer cell line CT26.WT and if OCs with the incorporated mutation behave like malignant cells. To achieve this, three lines of OCs in different conditions were exposed to CT26.WT cells through transwell co-culture for a week. The presence of *Kras*^*G12D*^ and capacity to form tumors were analyzed in treated samples by droplet digital PCR and colony-forming assays, respectively. The results showed that the *Kras*^*G12D*^ mutation was detected in hepatic culture conditions of undifferentiated OCs and these cells were capable of forming tumors in vitro. Therefore, OCs are susceptible to malignant transformation by horizontal transfer of DNA with *Kras*^G12D^ mutation in an undifferentiated condition associated with the liver microenvironment. This study contributes to a new step in the understanding of the colorectal metastatic process.

## Introduction

Colorectal cancer (CRC) is the third most common malignancy worldwide and the second leading cause of cancer death^1,2^. But the most typical CRC cause of death is not due to the main tumor, it is owing to metastases^3^. Around 50% of patients will develop liver metastasis being surgical resection the principal treatment, but a minority of patients are suitable for surgery^2^. Only 12%–14% of patients with metastatic CRC survive more than 5 years from diagnosis^4^. All this makes necessary a personalized treatment approach to improve the outcomes^2^. The circulation of tumor cells through the blood system has been proposed as a metastatic mechanism, but it is a highly inefficient process because the malignant cells must overcome many obstacles such as evading the immune system, infiltrating distant tissues, adapting to new niches and surviving to the host tissue replacement^5^. Additionally, most cells in the vascular torrent fail to form tumors at distant sites^6^. Therefore, other mechanisms could drive CRC metastasis that need to be explored. Thus, there is not an effective therapy against metastatic disease yet^3^.

In the last two decades, several studies have demonstrated the presence of circulating nucleic acids in the blood of CRC patients^7–9^ and the ability of this genetic material to transform susceptible cells by horizontal transfer of circulating cell–free DNA (cfDNA)^10^ (Genometastasis Theory)^7,11,12^. This capacity to transform cells can be tracked by mutations on *KRAS* that have been detected in susceptible cells treated with plasma or serum from CRC patients with *KRAS* mutations in the main tumor^7,8^. Around 35%–50% of human CRCs have a mutation of *KRAS*, in addition to inactivation of *APC* and *TP53*. The presence of mutations of *KRAS* is correlated with more aggressive disease, metastasis and the treatment with EGFR inhibitors is useless, targeting downstream signalling components of the *KRAS* pathway^4,9^. Mutations of *KRAS* promote not only the proliferation of cancer cells but also the infiltration of immunosuppressive cells such as regulatory T cells (Tregs)^13^. The most frequent mutation of *KRAS* is GAT (G12D)^4,13^, this mutation is present in mouse cell line CT26.WT which is the colon tumor animal model most widely used for targeted immunotherapy assessment and preclinical evaluation^14^.

In parallel, the liver receives 70–75% of the blood through the portal vein, which gets the entire splanchnic blood (25% from spleen and pancreas and 75% from the stomach and the intestines)^15^. This facilitates the constant influx of bioactive particles colon–liver that can participate in the metastatic process. Once the blood reaches the liver, it goes through the hepatic lobes to terminal branches of the intrahepatic biliary system, the canals of Hering, where there is a type of cells named hepatic progenitor cells (HPCs), oval cells in rodents (OCs). These cells are quiescent in the hepatic tissue under physiological conditions, but they emerge and expand from the portal triad when hepatocyte proliferation is overwhelmed^16^ by persistent and severe liver damage caused by carcinogens or hepatotoxins, hindering their isolation, particularly in humans. For this reason, animal models are more frequently used to isolated OCs and to study their involvement in liver disease^17^. Several authors^18–22^ have shown that OCs are implicated in hepatic malignancies and regarding the mutation of *Kras*^*G12D*^, this oncogene causes hepatic alterations, leading to liver tumorigenesis^23,24^. It should also be noted that the tumor microenvironment plays an important role too^25,26^, participating in cancer progression by alterations and degradation of extracellular matrix (ECM) components^27^ and activating the epithelial-mesenchymal transition (EMT), crucial for malignant development^28^.

For all these reasons, the aim of this study is to determine if OCs are susceptible to oncological transformation by treatment with CT26.WT cells harbouring a *Kras*^*G12D*^ mutation, generating an in vitro model, mimicking the mechanism of liver cell transformation by horizontal DNA transfer from colon cancer cells in culture.

## Results

### OCs express epithelial, hematopoietic, mesenchymal and hepatic markers

**Figure 1:**
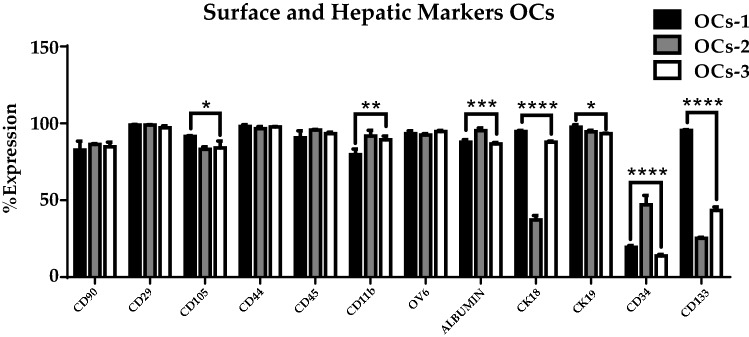
Percentage expression of surface and liver markers for three oval cell lines (OCs-1, OCs-2 and OCs-3) for characterization. Values are expressed as mean percentages with standard deviation (SD), one-way ANOVA test, *p < 0.05, **p < 0.01, ***p < 0.001, ****p < 0.0001, α = 0.05.

A flow cytometry analysis was performed to analyze OCs surface and hepatic markers. In OC-1, OC-2 and OC-3 lines, the most significant differences were found in markers CK18, CD34 and CD133. Furthermore, differences in CD105, CD11b, Albumin and CK19 were found when analysed by one-way ANOVA, P-value < 0.0001, α = 0.05 (Fig. 1). All these markers were more different in OCs-1 line compared to OCs-2 and OCs-3.

### OCs can differentiate into hepatocytes and into osteogenic and adipogenic lineages

**Figure 2:**
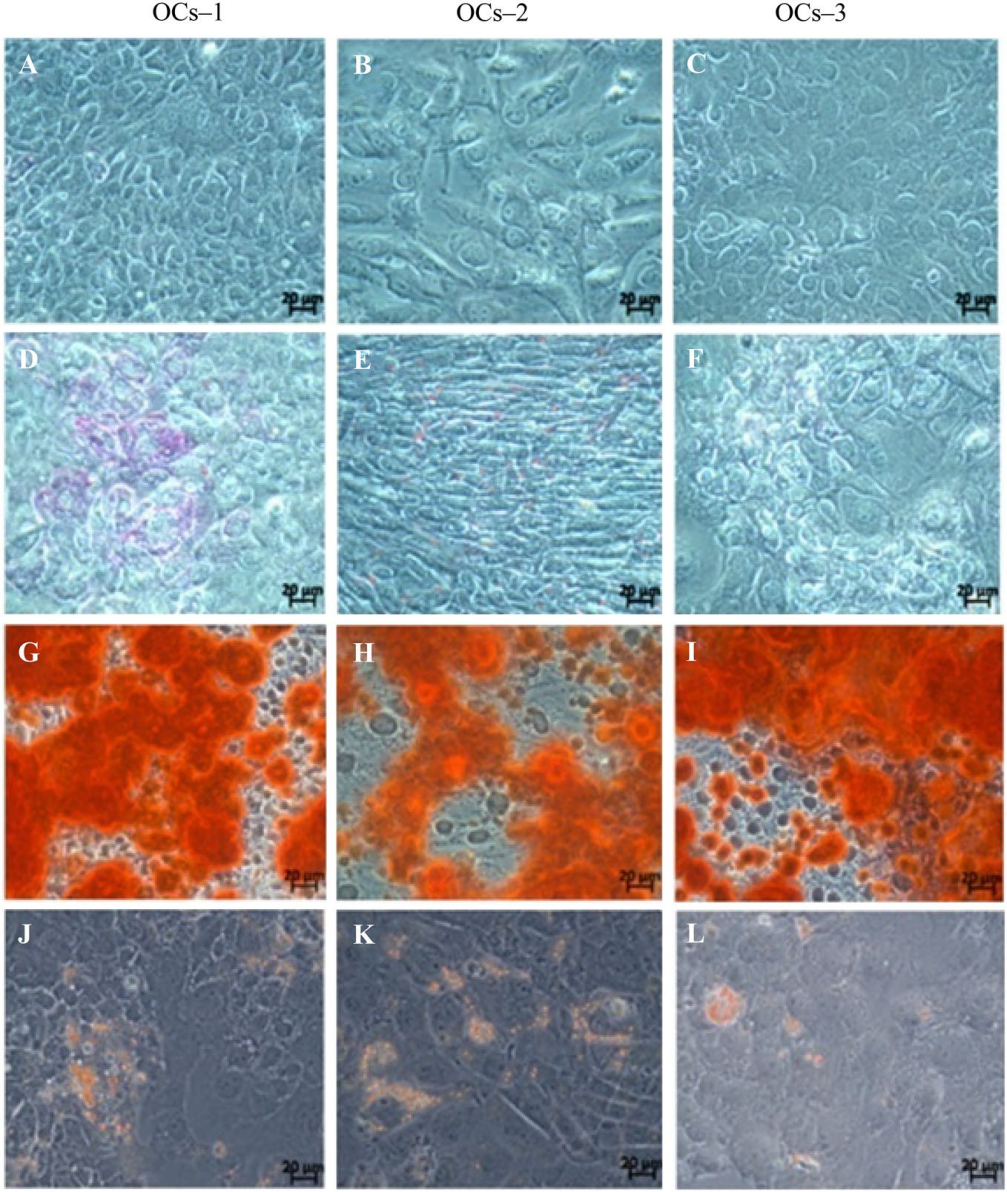
Differentiation towards hepatic, osteogenic and adipogenic lineages of three oval cell lines (OCs-1, OCs-2 and OCs-3). (**A**–**C**) Controls of OCs-1, OCs-2 and OCs-3, (**D**–**F**) PAS staining for liver differentiation, (**G**–**I**) Alizarin Red S staining for osteogenic differentiation and (**J**–**L**) Oil Red staining for adipogenic differentiation. Objective 63 ×.

The accumulation of glycogen in OCs was observed by PAS staining after hepatic differentiation treatment. This accumulation is greater in OCs-1 as compared to OCs-2 and OCs-3, being negligible in the latest (Fig. 2D–F). Compared to their controls (Fig. 2A–C), after differentiation treatment, all OC lines were able to accumulate salts (Fig. 2G–I) and lipids (Fig. 2J–L).

All these analyses confirm that OCs maintain their main characteristics along cell culture. Differences were found between OC lines analysed in terms of surface and hepatic markers and their capacity of differentiate into hepatocytes. The next step was to test whether these cells are susceptible to tumor transformation by co–culture with CT26.WT cells in transwell.

### Presence of *Kras*^*G12D*^ mutation in OCs-1 treated with CT26.WT cells

**Figure 3:**
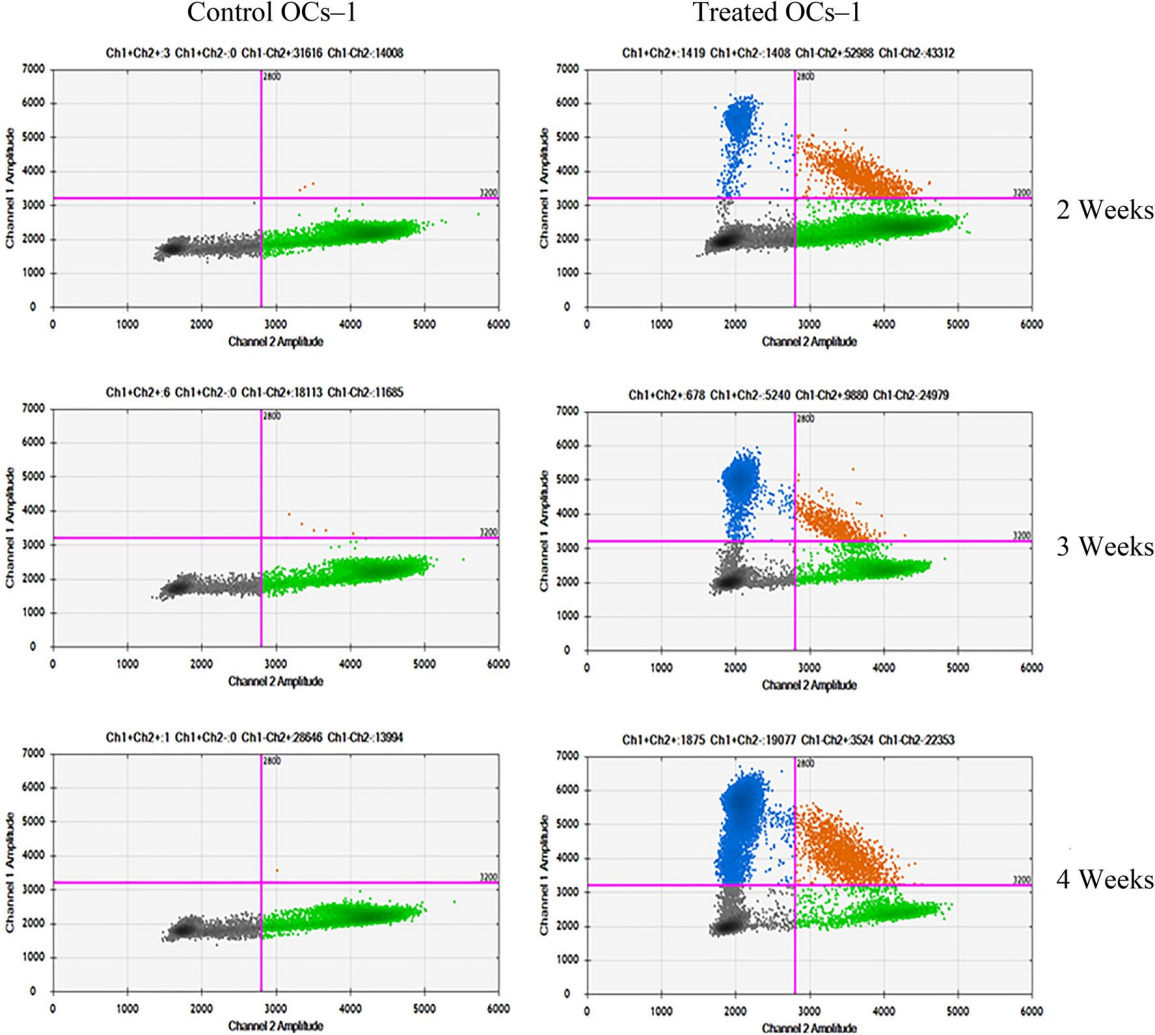
Droplet digital PCR (ddPCR) for the detection of mouse *Kras*^*G12D*^ in control OCs-1 and treated OCs-1 with CT26.WT cells to confirm the malignant transformation. Black points (droplets without *Kras* amplification), green points (droplets containing *Kras*^*WT*^ molecules), blue points (droplets containing amplified *Kras*^*G12D*^) and orange points (droplets containing both *Kras*^*WT*^ and *Kras*^*G12D*^ molecules).

First, the presence of *Kras*^*G12D*^ mutation in the conditioned medium of CT26 in transwell without OCs was confirmed (data not shown). The *Kras*^*G12D*^ mutation was detected in all OC lines tested, although OCs**-**2 and OCs-3 showed fewer mutated copies/µL (0.3 and 0.37 respectively) than OCs**-**1 (977 copies/µL) and the differences with their controls were less significant (Supplementary Table S1). The results show the presence of *Kras*^*G12D*^ in OCs-1 treated with CT26.WT cells in culture condition-1 to be the most significant and the mutation concentration is enhanced as the time in culture increases (Fig. 3). To find out whether the malignant transformation capacity is due to the cell line and/or culture conditions, we combined all OC lines with all culture conditions, but the *Kras*^*G12D*^ mutation was detected only in the primary culture condition of each OC lines (Conditions 1–3; Supplementary Table S1). It was also tested if the *Kras*^*G12D*^ OCs-1 were capable of transforming other healthy OCs but they did not show this capacity (Supplementary Table S2). OCs-1 maintained in culture conditions closer to liver microenvironment seem to be the cells more susceptible to malignant transformation than the other OC lines and therefore, the transformation study focused on these cells.

### The secretome is altered in *Kras*^*G12D*^ OCs-1

Next, the secretome of OCs-1 that have incorporated the *Kras*^*G12D*^ mutation from CT26.WT cells was analyzed as compared to their controls (*Kras*^*WT*^ OCs-1). It was found that the profile of cytokines present in the supernatant differed between these two conditions; specifically, differences were found in G-CSF, GM-CSF, IFN-γ, IL-3, IL-6, IL-12(p40), KC, MCP-1, MIP-1α, MIP-1β, RANTES and TNF-α (Table 1).

**Table 1:** Cytokines present in supernatants of OCs-1 *Kras*^*WT*^ and *Kras*^*G12D*^ OCs-1.

Cytokines	*Kras*^WT^ OCs-1	*Kras*^*G12D*^ OCs-1	*Kras*^WT^ OCs-1 vs * Kras*^*G12D*^ OCs-1	
	Mean ± SD (pg/mL)	Mean ± SD (pg/mL)	SIG.	Expression in *Kras*^*G12D*^ OCs-1
CCL11	25.28 ± 3.03	23.24 ± 2.27	NS	
G-CSF	144.77 ± 14.15	67.32 ± 7.94	****	Down-regulated
GM-CSF	61.94 ± 2.77	35.76 ± 3.37	****	Down-regulated
IFN-γ	2.16 ± 0.43	2.78 ± 0.62	*	Up-regulated
IL-1α	2.11 ± 0.28	1.92 ± 0.19	NS	
IL-1β	1.94 ± 0.60	1.98 ± 0.68	NS	
IL-2	0.65 ± 0.39	0.97 ± 0.39	NS	
IL-3	1.67 ± 0.25	1.43 ± 0.16	*	Down-regulated
IL-4	0.65 ± 0.58	0.52 ± 0.38	NS	
IL-5	1.47 ± 0.16	1.47 ± 0.34	NS	
IL-6	0.92 ± 0.14	2.5 ± 0.18	****	Up-regulated
IL-9	2.66 ± 0.28	2.83 ± 0.33	NS	
IL-10	2.73 ± 1.37	3.04 ± 0.61	NS	
IL-12(p40)	16.50 ± 1.73	14.41 ± 1.36	*	Down-regulated
IL-12(p70)	4.26 ± 3.03	5.70 ± 5.72	NS	
IL-13	13.76 ± 6.46	10.69 ± 4.83	NS	
IL-17A	0.69 ± 0.40	1.10 ± 1.17	NS	
KC	2489.24 ± 555.04	824.04 ± 101.65	****	Down-regulated
MCP-1	490,224 ± 233,447	92,144 ± 21,602	***	Down-regulated
MIP-1α	0.29 ± 0.07	0.56 ± 0.08	****	Up-regulated
MIP-1β	6.68 ± 0.63	12.15 ± 0.76	****	Up-regulated
RANTES	28.17 ± 2.90	1537.01 ± 141.94	****	Up-regulated
TNF-α	18.76 ± 1.70	16.59 ± 1.42	**	Down-regulated

*Student’s *t*-test, *p < 0.05, **p < 0.01, ***p < 0.001, ****p < 0.0001 and NS = not significant, α = 0.05. Interleukins: IL-1α, IL-1β, IL-2, IL-3, IL-4, IL-5, IL-6, IL-9, IL-10, IL-12 (p40), IL-12 (p70), IL-13, IL-17A, Eotaxin (CCL11), Granulocyte-Colony Stimulating Factor (G-CSF), Granulocyte Macrophage-Colony Stimulating Factor (GM-CSF), Interferon-γ (IFN-γ), Keratinocyte Chemoattractant (KC), Monocyte Chemoattractant Protein-1 (MCP-1), Macrophage Inflammatory Protein-1α (MIP-1α), Macrophage Inflammatory Protein-1β (MIP-1β), RANTES (CCL5) and Tumor Necrosis Factor-α (TNF-α).

### The surface and hepatic markers of *Kras*^*G12D*^ OCs-1 present alterations

**Figure 4:**
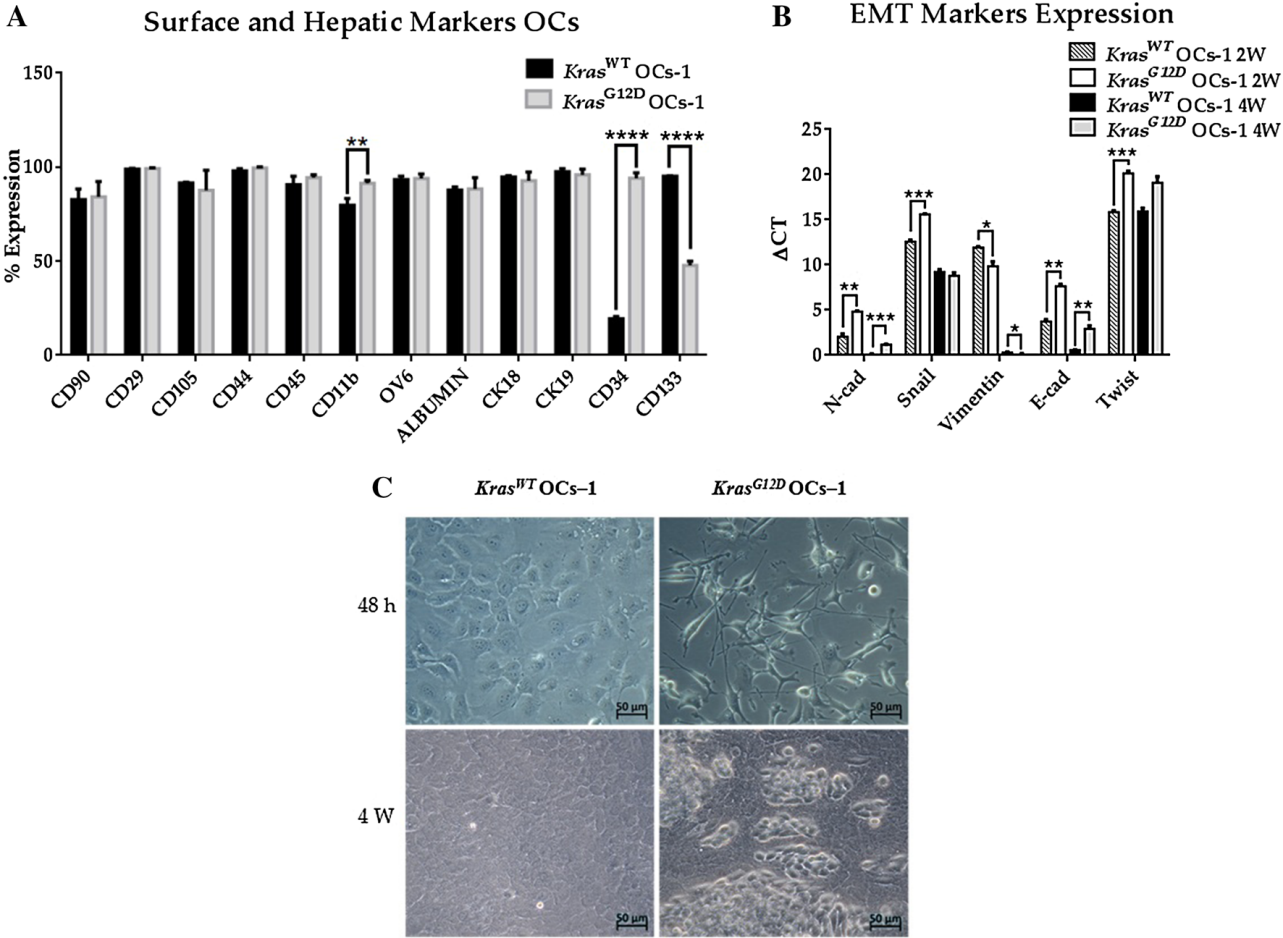
Changes observed in *Kras*^*G12D*^ OCs-1 with respect to *Kras*^*WT*^ OCs-1. Three replicates of *Kras*^*WT*^ OCs-1 and *Kras*^*G12D*^ OCs-1, Student’s *t*-test *p < 0.05, **p < 0.01, ***p < 0.001, ****p < 0.0001, α = 0.05. (**A**) Mean of expression percentages and SD of hepatic and surface markers. (**B**) Variation of gene expression of mouse EMT markers (N-Cadherin, Snail, Vimentin, E-Cadherin and Twist) between 2 and 4 weeks of culture (2 W and 4 W respectively) analyzed by RT-qPCR normalized to *Gapdh* housekeeping gene. (**C**) Morphology changes observed at optical microscopy (O.M) at 48 h and 4 weeks of culture. Objective 40 ×.

The analysis of surface markers of *Kras*^*G12D*^ OCs-1 revealed that most of them did not change as compared to OC-1 *Kras*^WT^: differences were only found in CD11b, CD34 and CD133 markers (Fig. 4A).

### There are changes in EMT markers expression of *Kras*^*G12D*^ OCs-1

Significant differences were found in EMT markers of OCs-1 treated with CT26.WT cells in comparison with *Kras*^*WT*^ OCs**-**1 after treatment (2 weeks) and with *Kras*^*WT*^ OCs**-**1 at 4 weeks of culture (Fig. 4B). The evolution of EMT markers in *Kras*^*G12D*^ OCs**-**1 over time, shows a transition towards mesenchymal phenotype. Also, a morphological change was detected: *Kras*^*G12D*^ OCs-1 acquired a fibroblastic shape, whereas *Kras*^*WT*^ OC**-**1 presented a polygonal morphology (Fig. 4C).

### *Kras*^*G12D*^ OCs-1 proliferate more than *Kras*^*WT*^ OCs-1

**Figure 5:**
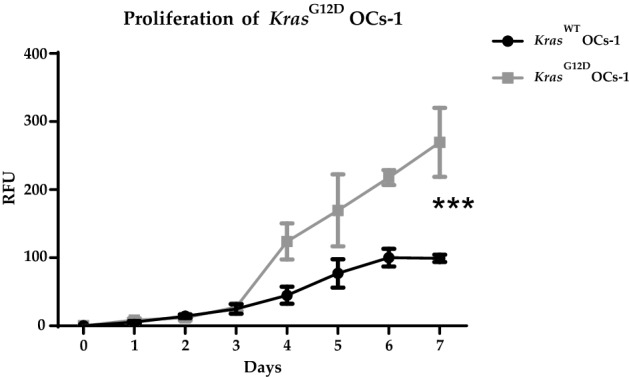
Alamar Blue assay for the proliferation of *Kras*^*WT*^ OCs-1 and *Kras*^*G12D*^ OCs-1. Mean and SD of three replicates of *Kras*^*WT*^ OCs-1 and *Kras*^*G12D*^ OCs-1. Relative fluorescence units (RFU). Student’s *t*-test *** *p*-value = 0.0003, (α = 0.05).

To test if the *Kras*^*G12D*^ OCs-1 have acquired higher proliferative capacity with respect to *Kras*^*WT*^ OCs-1, an Alamar Blue assay was performed. *Kras*^*G12D*^ OCs-1 were compared with *Kras*^*WT*^ OCs-1 and significant differences were found after day 4 of the assay by Student’s *t*-test *p*-value = 0.0003, α = 0.05 (Fig. 5).

### *Kras*^*G12D*^ OCs-1 are capable of forming tumors in vitro

**Figure 6:**
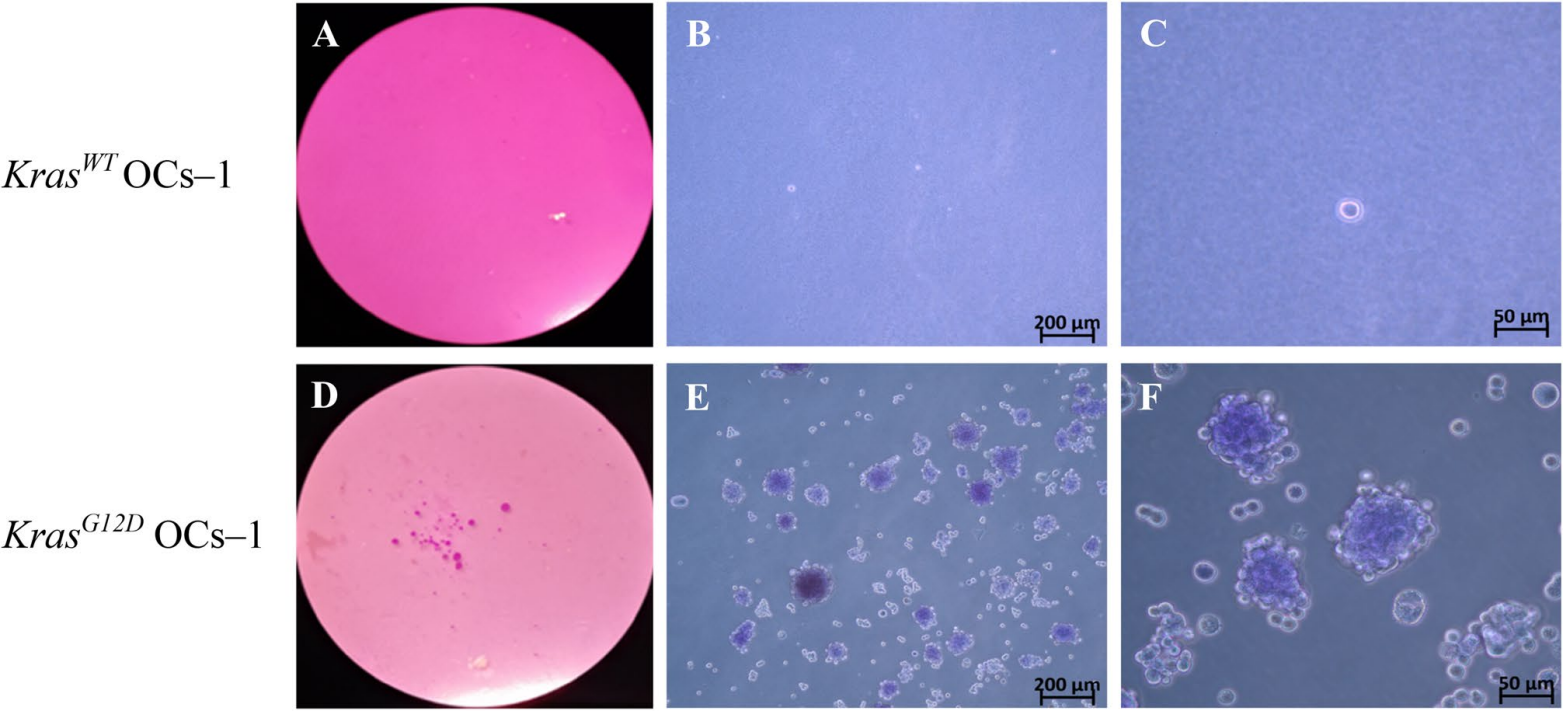
Noble Agar assay for *Kras*^*WT*^ OCs-1 (**A**–**C**) versus *Kras*^*G12D*^ OCs-1 (**D**–**F**). Photos by Loupe, Objective 16 × (**A** and **D**) and optical microscope (O.M), objectives 10 × (**B** and **E**) and 40 × (**C** and **F**).

The *Kras*^*G12D*^ OCs-1 were able to form colonies on agar while *Kras*^*WT*^ OCs-1 were not. As Fig. 6 shows, after 21 days in culture, *Kras*^*WT*^ OCs-1 remained as isolated cells because they were not able to proliferate in Noble Agar (Fig. 6A–C), whereas the *Kras*^*G12D*^ OCs-1 were able to form colonies from isolated cells (Fig. 6D–F).

## Discussion

New approaches are needed to understand the hepatic metastasis process of colorectal cancer. OCs have been shown to be involved in various liver neoplasms^18–21^. For this reason, the present study focused on the malignant transformation susceptibility of OCs and offers a possible in vitro pathway of liver metastasis of colorectal cancer through the incorporation of the *Kras* GAT (G12D) oncogene by horizontal transfer. This mutation was chosen for this research because it is the most frequent in colorectal cancer, the most aggressive, and with the worst prognosis^4^ and it has also been implicated in the development of liver tumors such as cholangiocarcinoma and hepatocellular carcinoma (HCC)^23,24^. Recent studies have shown that microvesicles with nucleic acids from cancer cells can contribute to horizontal transfer of oncogenes and they are associated with transforming phenotype and modulation of the microenvironment for metastatic spread^29–32^. The tumor-derived microvesicles uptake by organ-specific cells prepares the pre-metastatic niche^33^. In this way, the incorporation of *Kras*^*G12D*^ via microvesicles has been demonstrated in mouse fibroblasts^7^ but not in hepatic progenitor cells yet.

The OCs are heterogeneous, constituting a spectrum of cells ranging from an immature phenotype to mature cholangiocytes (CK19) and intermediate hepatocytes (CK18, Albumin). Moreover, they share common characteristics with cells of hematopoietic system (CD105, CD34, CD11b, CD133)^34–38^. Differences were found in these markers between the OC lines tested in the current study. This work shows that *Kras*^*G12D*^ was detected in OCs-1 when these cells were exposed without cell contact with CT26.WT cells in an enriched culture closer to liver microenvironment. Since the expected incorporation of *Kras*^*G12D*^ in OC-1 cells was very low^7^, we decided to perform a ddPCR, which presents a higher sensitivity and accuracy of detection than other methods^39–43^. OCs-1 incorporated this oncogene in a stable way, while OCs-2 and OCs-3 were less susceptible. It seems that OC lines (OCs-2 and OCs-3) with a phenotype less committed to hepatocytes and closer to hematopoietic identity were less susceptible to malignant transformation. In contrast, the OCs-1 phenotype was closer to progenitor cells and showed more differences in surface and hepatic markers compared to OCs-2 and OCs-3. Also, the culture medium of OCs-1 was more similar to the hepatic microenvironment, in contrast to OCs-2 and OCs-3 culture conditions. In any event, the specific culture conditions are not enough for these cells to transform, since when cultivating OCs-2 and OCs-3 in the same medium as OC-1, the *Kras*^*G12D*^ mutation was not detected.

Additionally, the presence of *Kras*^*G12D*^ altered the immune response, inducing immunosuppressant cytokines and/or enhancing the inflammatory response^13,44,45^. It was found that in *Kras*^*G12D*^ OCs-1, G-CSF, GM-CSF, IL-3, IL-12 (40p), KC, MCP-1 and TNF-α were down-regulated, whereas INF-γ, IL-6, MIP-1α, MIP-1β and RANTES were up-regulated. Interestingly, persistent activation of the IL-6 signalling pathway might result in the development of liver tumors^26,46^ and RANTES or CCL5 are overexpressed in chronic liver disease and implicated in tumor initiation and progression especially in HCC^47^. Changes in EMT related genes were also detected. This may be associated with the EMT alteration, implicated in cancer with poor prognosis and metastases^48,49^. In this study it was found that epithelial marker such as E-cadherin was up-regulated over time in *Kras*^*G12D*^ OCs-1. These findings are consistent with the high expression of epithelial markers CK18 and CK19 found in these cells. The mesenchymal markers (N-cadherin, Snail, Vimentin) also were up-regulated over time. These results seem to indicate that the transformed OCs are in a transitional phase between epithelial and mesenchymal phenotype. In agreement with this, Christiansen and Rajasekaran previously showed that advanced carcinomas adopt some mesenchymal features while retaining characteristics of well–differentiated epithelial cells^50^. It is worth to mention that CD133 plays an important role in facilitating the EMT regulatory loop, in particular by up–regulating the expression of N-cadherin^51^. Importantly, the mesenchymal–epithelial transition (MET) is implicated in appropriate features required to prepare the soil as premetastatic niche and promote tumor progression, while EMT remodeling, via Wnt y β-catenin is altered in liver^52^.

On the other hand, OCs share many characteristics with endothelial cells, epithelial cells, mesenchymal and hematopoietic stem cells. For this reason, a broad panel of surface markers recommended by several authors^34–36^ has been analyzed in this study. Such analysis revealed alterations in surface markers in *Kras*^*G12D*^ OCs-1 in comparison with *Kras*^*WT*^ OCs-1. While there was down-regulation of progenitor cell marker CD133^37,53^, the surface markers CD11b and CD34 were overexpressed. These markers were not altered in OC-2 with mutated *Kras* despite having the same origin as OC-1, a possible explanation for this might to be that the different baseline expression of these markers between OCs-1 and OCs-2. Also, CD11b had not previously been described as an OCs marker but it seems feasible taking into account that OCs share common characteristics with hematopoietic system^34^. Interestingly, the OCs were positive for CD11b and *Kras*^*G12D*^ OCs-1 presented overexpression on this marker. Maybe pro-inflammatory stimuli such as IFN-γ enhanced CD11b surface expression^54^. An association between *Kras*^*G12D*^ and the enhanced expression of CD11b has been found in mouse^55^ but more investigation in this field is necessary to reach a more definitive conclusion. At the same time, liver cancer cells are CD34 positive^56^, for this reason CD34 may be contributing to the metastatic process too.

Finally, to test whether OCs with *Kras*^*G12D*^ have a malignant behaviour, the Alamar Blue^57^ and Noble Agar^58^ assays were performed. Tumor cells commonly show a high proliferative ability as well as the ability to form colonies on Noble Agar whereas untransformed cells do not^59^.

A better understanding of the malignant transformation of liver cells, by horizontal DNA transfer from CT26.WT cells (mutant *Kras*^*G12D*^ as reporter), would be helpful to develop more effective and targeted therapies against CRC metastases. Despite the limitations of this study, for example the use of 2D cultures instead 3D cultures, the findings are consistent with the hypothesis that OCs are susceptible to malignant transformation by the cell free DNA bearing *Kras*^*G12D*^ oncogene. The results presented in this manuscript have proved that *Kras*^*G12D*^ OCs have higher proliferation rate than OCs without *Kras*^*G12D*^ and have acquired the ability to form colonies in Noble Agar, which means that primary OCs can be transformed with *Kras*^*G12D*^ under enriched culture conditions mimicking the hepatic microenvironment.

## Methods

### Oval cells (OCs)

Three OC lines were kindly provided by Aránzazu Sánchez, School of Pharmacy, Universidad Complutense de Madrid (UCM), Madrid, Spain. To obtain OCs, 9-week-old C57BL/6 male Met^flx/flx^ or wild-type (WT) mice were maintained on 0.1% 3,5-diethoxycarbonyl-1,4-dihydrocollidine (DDC-supplemented diet) for 13 days and then the OCs-enriched non-parenchymal cell fraction was isolated and plated. OCs were selected based on their characteristic epithelial morphology and subcultured for further expansion and characterization. Once established, these cell lines were phenotypically and functionally characterized and validated^20,60^. Three OC lines were used in this study, OCs-1 and OCs-2 derived from WT mice and OCs-3 derived from Met^flx/flx^ mice. OCs-3 are a Met^flx/flx^ OC cell line, we took advantage of them from other research^20^ and was generated from mice homozygous for the Met floxed allele, a conditional knockout mouse for c-Met generated using the Cre-loxP-mediated gene targeting system^61^. The Met inactivation by infecting in vitro the parental Met^flx/flx^ OC line with an adenovirus, have not been used in the present work, we used Met^flx/flx^ OCs (corresponding to OCs-3), they express a normal functional Met receptor. Besides, the Met^flx/flx^ OC line has been used in a number of additional studies having proved to have an intact Met signaling and to behave as WT OCs^60,62–64^ and because of this, the OCs-3 have been included in the present research. All OC lines were harvested at 80%–90% confluence using Tripsin-EDTA and replated at 0.5–1 × 10^4^ cells/cm^2^ with two changes of medium per week until their use for the experiments. Only early passage cells (passages 4–6) were used. Different culture conditions were used for each OC line provided (Supplementary Table S3) Condition 1: closer to liver microenvironment, condition 2: intermediate condition between 1 and 3 and condition 3: standard culture conditions.

### Mouse colon cancer cell line CT26.WT

This ATCC cell line was kindly provided by Miguel Urioste Azcorra from Spanish National Cancer Research Centre (CNIO), Madrid, Spain. CT26.WT was chosen because it has the *Kras*^*G12D*^ mutation. The cells were maintained with Dulbecco’s Modified Eagle Medium (DMEM). Cells were harvested at 80%–90% confluence using Tripsin–EDTA and replated 0.5–1 × 10^4^ cells/cm^2^ with two changes of media per week until use for the experiments.

OCs lines and CT26.WT cells were cultured with 1% ZellShield (Minerva Biolabs, Berlin, Germany) and 10% Fetal Bovine Serum (FBS) and incubated at 37 °C, 5% CO_2_.

### Transwell co-culture

For the malignant transformation experiments, 6-well transwell culture plates (Corning, Ref.: 3450) with 0.4 µm membrane pore were used. In the lower chamber of the transwell, 3 × 10^4^ cells of each OC line were seeded separately and 2 × 10^4^ CT26.WT cells were seeded in the upper chamber of the transwells. Previously, the wells at the bottom of the transwell with OCs-1 and OCs-2 were precoated with collagen type I. The experiments were repeated three times and OCs co-cultured with OCs from the same line were used as controls. Treatments with CT26.WT cells were maintained for a week incubated at 37 °C, 5% CO_2_, with two corresponding changes of medium. After a week, the treatments were withdrawn and the sample supernatants were collected. Cells were harvested and 1.2 × 10^4^ cells/cm^2^ were seeded again at two, three, and four weeks for further analysis. To elucidate whether the transformation is due to OCs and/or culture conditions, after this study all cells were maintained under all other culture conditions (1, 2 and 3). These conditions were useful to test the microenvironment effect on malignant transformation by *Kras*^*G12D*^ from CT26.WT cells. It was also tested if the transformed OCs were capable of transforming other healthy OCs. All these last experiments were performed with the transwell protocol previously mentioned.

### Alamar blue assay

This assay was performed according to the manufacturer protocol (Invitrogen, Eugene, OR, USA) to compare the proliferation between each OC line and the treated OCs against their respective controls in transwell. Each OC line was seeded at 1.3 × 10^3^ cells/cm^2^ in 12-well culture plates. At 24 h, Alamar Blue (10%) was added to the culture medium and incubated at 37 °C and 5% CO_2_ for 2 h. After incubation, 100 µL of medium of each sample were transferred to 96 well plates and fluorescence was read (560 nm Excitation/590 nm Emission) on an EnSpire multimode Plate Reader (Perkin Elmer, Waltham, MA, USA) with Enspire Manager Software Version 4.

### Flow cytometry

For the characterization of OCs and the analysis of changes in surface and protein markers of OCs treated with CT26.WT cells, flow cytometry was performed. OCs were harvested using Trypsin EDTA (1X) and they were resuspended in 100 µL of cold Phosphate Buffered Saline (PBS) at a density of 1 × 10^5^ cells per tube. The cells were incubated 30 min at 4 °C, in dark conditions with the following mouse primary antibodies (mAbs) CD11b, CD90, CD29, CD34, CD45, CD44, CD105, CD133, OV6, Albumin, CK18, CK19 according to each manufacturer instructions (Supplementary Table S4). After immunostaining, cells were rinsed with PBS and they were centrifuged at 200×*g* for 5 min. Then, the cells were acquired by Fast Canto II cytometer (Becton Dickinson, Franklin Lake, NJ, USA). The results were analyzed with FlowJo Software Version 10.

### Differentiation assay

As progenitor cells, the OCs are able to differentiate into hepatocytes^65^ and share many characteristics with mesenchymal stem cells^66^. Therefore, for a more detailed characterization of these cells it has been considered appropriate to differentiate them towards hepatocyte, adipocyte and osteocyte lineages:

#### Hepatocyte differentiation

To differentiate OCs into hepatocytes^67,68^, 1 × 10^5^ cells/well were seeded in a six–well plate under the corresponding culture conditions for each OC line. The following day, dimethyl sulfoxide (DMSO) (1%) was added to culture medium and it was maintained for four days. Then, the culture medium with DMSO was removed and fresh culture medium with sodium butyrate (2.5 mM) was added. This condition was maintained for six days with medium replacement twice a week. Finally, the medium with sodium butyrate was removed and fresh medium with HGF (10 ng/mL) was added. This new condition was maintained for six days. At the end of this experiment, the presence of hepatocytes was analyzed by Periodic acid–Shiff staining (PAS) (Sigma-Aldrich, St. Louis, MO, USA) according to the manufacturer's instructions.

#### Osteogenic differentiation

Mouse Mesenchymal Stem Cell Functional Identification Kit (R&D, Minneapolis, MN, USA) with osteogenic supplement was used according to the manufacturer instructions for culture during 28 days with culture medium replacement twice a week. After 28 days, OCs cultures were rinsed with distilled water (H_2_O_d_) and fixed with cold 70% ethanol for 1 h at room temperature (RT). Then, the ethanol was removed and cells were rinsed with H_2_O_d_. The calcium deposits were stained with Alizarin Red S (Sigma-Aldrich) (0.01 g/mL) for 1 h at RT and then the cultures were rinsed with H_2_O_d_ to remove the excess stain.

#### Adipogenic differentiation

The OCs were treated with differentiation Mouse Mesenchymal Stem Cell Functional Identification Kit (R&D) with adipose supplement according to the manufacturer instructions for 14 days with culture medium replacement twice a week. After 14 days, the cultures were rinsed with PBS and fixed with 10% formaldehyde for 30 min. Then the cells were rinsed again with PBS and fixed with 60% of isopropanol for 5 min. The isopropanol was removed and the lipid droplets accumulated were stained with Oil Red O (Acros Organics, NJ, USA) (3 mg/mL in 36% isopropanol) for 1 h at RT. Finally, the cells were washed with 60% isopropanol to remove excess of stain and rinsed with PBS.

All differentiation experiments had their corresponding controls with conventional culture medium and pictures were taken for each OC line by optical microscopy (Zeiss Axio Vert A.1, Palex Medical, Madrid, Spain) and Image Software Zen 3.1.

### DNA isolation and droplet digital PCR (ddPCR) for mouse *Kras*^*G12D*^ mutation detection

After transwell experiments, the DNeasy Blood & Tissue Kit (Qiagen, Hilden, Germany) was used for the extraction of total DNA from OCs samples and it was quantified by fluorimetry in a Qubit® 2.0 Fluorometer using the Qubit™ dsDNA BR Assay Kit (Thermo Fisher Scientific, Waltham, MA, USA).The presence of *Kras*^*G12D*^ was analysed by a custom droplet digital PCR (ddPCR) assay using the QX200 Droplet Digital PCR System (Bio-Rad Laboratories, Hercules, CA, USA). Samples were prepared by mixing 10 μL of ddPCR Supermix for probes (No dUTP, Bio–Rad), 1 μL of HindIII restriction enzyme (5 U/μL) (Thermo Fisher Scientific), 1 μL of FAM and HEX fluorescent probes (specific for mutant *Kras* and wild-type *Kras*, respectively) (Supplementary Table S5A), and 1 to 6 μL of template DNA in a final reaction volume of 20 μL. A total amount of 100 ng of cell-derived DNA was added per well. Three replicates were analysed per sample. Water instead of DNA was used for no template control (NTC) and served as a control for detecting environmental contamination. Genomic DNA from each OC line was used as a negative control to estimate the false-positive rate; and a positive control containing genomic DNA from the *Kras*^*G12D*^ mouse colon carcinoma cell line CT26.WT was used to verify the assay performance and determine the threshold value of fluorescent signals. Droplets were generated by a QX200 droplet generator (Bio-Rad) and endpoint PCR was performed on a T100 Thermal Cycler (Bio-Rad). After thermal cycling (Supplementary Table S5B), the fluorescent signals of droplets were detected in the FAM and HEX channels of a QX200 droplet reader (Bio-Rad).The ddPCR data were analyzed using Quanta Soft v.1.7 Software (Bio-Rad). Results were reported as the number of copies of *Kras*^*G12D*^ per μL/reaction. Poisson distribution was used to determine the concentration of *Kras*^*G12D*^*.* To determine if one sample is positive for this mutation, the concentration of *Kras*^*G12D*^ (copies/µL reaction) in the merged replicates of each sample was compared with a wild-type control (of similar WT concentration) using a Z-test and assuming that concentrations follow a normal distribution.

### Cytokine assay

After treatment with CT26.WT cells for a week, the supernatants of different OCs were collected in 15 mL tubes. The tubes were centrifuged at 200×*g*, 15 min, 4 °C, the pellets were removed and the supernatants were centrifuged again, at 9.3 × 10^3^ g, 10 min, 4 °C, to remove any remaining cells and debris. The supernatants were aliquoted and frozen in 1.5 mL tubes at – 80 °C until use. For the analysis of cytokines present in supernatants of OCs, Bio-Plex Pro Mouse Cytokine 23-plex Assay (Bio-–Rad) was used according to manufacturing recommendations. The cytokines were read on a MAGPIX system with MILLIPLEX® Analyst 5.1 software (Merck Millipore). A Student’s *t*-test was performed to analyze the differences between the control and treated cells and all samples were analyzed in triplicate.

### RNA isolation and qRT–PCR for epithelial–mesenchymal transition (EMT) gene expression

NZY total RNA isolation kit (NZYTech, Lisboa, Portugal) was used for total RNA extraction and it was quantified by Nano Drop (Thermo Fisher Scientific). One μg of RNA was used to generate cDNA using High-Capacity cDNA Reverse Transcription Kit (Thermo Fisher Scientific, Baltics, UAB). The quantitative real-time PCR (qRT-PCR) was performed using GoTaq qPCR Master Mix (Promega, Madison, WI, USA) according to manufacturer instructions. The cDNA samples were run on a Thermal Cycler 7500 fast Real-Time PCR System (Applied Biosystems, Foster City, CA, USA). The relative expression gene method (2^–ΔCt^) and cycle threshold (Ct) were used as reference to analyze the results. Ct values were processed and normalized to glyceraldehyde 3-phosphate dehydrogenase (*Gapdh*), to account for the total amount of RNA in each sample. The samples were run in triplicate and the qRT-PCR primers were obtained from Gene Bank (Supplementary Table S6A,B) for mouse EMT and *Gapdh* genes expression.

### Noble agar assay

A 6–well plate was precoated with Noble Agar (1%) in conventional medium and when it was polymerized, the OCs (control and treated) were seeded at 5 × 10^3^ per well in medium with 0.6% Noble Agar. The cells were maintained at 37 °C and 5% CO_2_ in the incubator during 21 days adding 1 mL of medium each four days. Then, the results were analysed by optical microscopy (Zeiss) and binocular loupe (Leica Microsystems, Milan, Italy).

### Statistical analysis

GraphPad Prism version 6 was used for statistical tests. All assays were performed with three biological and technical replicates. To compare the proliferation (Alamar Blue) of *Kras*^*G12D*^ OCs versus *Kras*^*WT*^ OCs the results were analyzed by Mann Whitney test of Student´s *t*-test. One-way ANOVA was used for comparison of surface markers of OCs. Finally, to compare changes in surface markers, in cytokines and EMT markers, a Student’s *t*-test was used. The ddPCR results were analyzed with Z-test. In all experiments the *p*-value was considered statistically significant if lower than 0.05 with α = 0.05.

## Supplementary Information

Supplementary Information.
