# Supplementary material for: Oncological transformation in vitro of hepatic progenitor cell lines isolated from adult mice
Source: Sci Rep. 2022 Feb 24;12:3149. doi: 10.1038/s41598-022-06427-w (PMC8873244; doi:10.1038/s41598-022-06427-w)
Supplement: Supplementary file 1 — Supplementary Information. [file 41598_2022_6427_MOESM1_ESM.docx]

**Supplementary Information**

Oncological Transformation in Vitro of Hepatic Progenitor Cell Lines Isolated from Adult Mice

Rocío Olivera–Salazar^1^, Mariano García–Arranz^1,2^, Aránzazu Sánchez^3^, Susana Olmedillas–López^1^, Luz Vega–Clemente^1^, Luis Javier Serrano^1^ , Blanca Herrera^3^, Damián García–Olmo^1,2,4^.

^1^New Therapies Laboratory, Health Research Institute–Fundación Jiménez Díaz University Hospital (IIS–FJD), Avda. Reyes Católicos, 2, 28040, Madrid, Spain.

^2^Department of Surgery, School of Medicine, Universidad Autónoma de Madrid (UAM), Arzobispo Morcillo, 4, 28029, Madrid, Spain.

^3^Department of Biochemistry and Molecular Biology, School of Pharmacy, Universidad Complutense de Madrid (UCM), Plaza de Ramón y Cajal, s/n, 28040, Madrid, Spain.

^4^Department of Surgery, Fundación Jiménez Díaz University Hospital (FJD), Avda. Reyes Católicos, 2, 28040, Madrid, Spain.

*Correspondence: [olivera.rocio@hotmail.es](mailto:olivera.rocio@hotmail.es)

**Supplementary Table S1.** ddPCR results for *Kras^G12D^* presence in OCs after treatment with CT26.WT cells by transwell.

| Sample | Culture condition | Treatment | Positive events | Copies/µl | Signification |
| --- | --- | --- | --- | --- | --- |
| OC–1 | 1 | Untreated | 1 | 0,03 | **** |
|  |  | CT26.WT | 26271 | 977 |  |
|  | 2 | Untreated | 2 | 0,12 | NS |
|  |  | CT26.WT | 0 | 0 |  |
|  | 3 | Untreated | 1 | 0,06 | NS |
|  |  | CT26.WT | 2 | 0,06 |  |
| OC-2 | 1 | Untreated | 2 | 0,07 | NS |
|  |  | CT26.WT | 2 | 0,09 |  |
|  | 2 | Untreated | 0 | 0 | * |
|  |  | CT26.WT | 13 | 0,3 |  |
|  | 3 | Untreated | 1 | 0,05 | NS |
|  |  | CT26.WT | 2 | 0,09 |  |
| OC-3 | 1 | Untreated | 2 | 0,08 | NS |
|  |  | CT26.WT | 0 | 0 |  |
|  | 2 | Untreated | 2 | 0,11 | NS |
|  |  | CT26.WT | 0 | 0 |  |
|  | 3 | Untreated | 2 | 0,08 | * |
|  |  | CT26.WT | 7 | 0,37 |  |

*Comparison OCs untreated (Control) with OCs CT26.WT (Treatment), *Z*-test, * *p* < 0.05, **** *p* < 0.0001 and NS = not significant, α=0.05.

**Supplementary Table S2.** ddPCR results for *Kras^G12D^* presence in healthy OCs after treatment with *Kras^G12D^* OC–1 cells by transwell.

| Sample | Culture condition | Treatment | Positive events | Copies/µl | Signification |
| --- | --- | --- | --- | --- | --- |
| OC-1 | 1 | Untreated | 2 | 0,06 | NS |
|  |  | *Kras^G12D^* OC–1 | 0 | 0 |  |
| OC-2 | 2 | Untreated | 4 | 0,11 | NS |
|  |  | *Kras^G12D^* OC–1 | 1 | 0,04 |  |
| OC-3 | 3 | Untreated | 4 | 0,12 | NS |
|  |  | *Kras^G12D^* OC–1 | 3 | 0,09 |  |

* NS = not significant, *Z*-test, α=0.05.

**Supplementary Table S3.** Culture conditions asociated to each line of OCs.

| Culture condition | Associated  Cells | Culture  Medium | Precoated dishes Collagen type I (50 µg/mL) | Enriched |
| --- | --- | --- | --- | --- |
| Condition 1:  closer to liver microenvironment | OCs-1 | William´s E | Yes | Dexametasone (100Nm), epithelial growth factor (EGF) (10 ng/mL), hepatic growth factor (HGF) (10 ng/mL) and insulin–transferrin–selenium–ethanolamine (ITS) (1X). |
| Condition 2: intermediate  condition between 1 and 3 | OCs-2 | DMEM | Yes | No |
| Condition 3: standard culture condition | OCs-3 | DMEM | No | No |

**Supplementary Table S4.** Mouse antibodies for flow cytometry used for characterization OCs.

| Marker | Fluorocrome | Comercial |
| --- | --- | --- |
| CD11b | APC | Biolegend, San Diego, CA, USA |
| CD90 | FITC | Biolegend, San Diego, CA, USA |
| CD29 | FITC | Invitrogen, San Diego, CA, USA |
| CD34 | FITC | Invitrogen, San Diego, CA, USA |
| CD45 | PE | Invitrogen, San Diego, CA, USA |
| CD44 | PE–Cy7 | Invitrogen, San Diego, CA, USA |
| CD105 | APC | Invitrogen, San Diego, CA, USA |
| CD133 | APC | Invitrogen, San Diego, CA, USA |
| OV6 | PE | Santa Cruz, Dallas, TX, USA |
| Albumin | FITC | MyBioSource, San Diego, CA, USA |
| CK18 | FITC | Thermo Fisher Scientific, Regensburg, Germany |
| CK19 | FITC | Thermo Fisher Scientific, Regensburg, Germany |

*Allophycocyanin (APC), Fluorescein Isothiocyanate (FITC), R–phycoerythrin–Cyanine 7 (Pe–Cy7) or Phycoerythrin (PE).

**Supplementary Table S5.** **(A)** ddPCR Custom primers for mouse *Kras^WT^* and *Kras^G12D^* mutation. **(B)** ddPCR thermal cycling conditions for mouse *Kras^WT^* and *Kras^G12D^*.

**A**

| Assay | Forward / Reverse | Probe sequence | Dye |
| --- | --- | --- | --- |
| Mut | 5´–GGCCTGCTGAAAATGACTGA–3´  3´–AGCAGCGTTACCTCTATCGT–5´ | TGCCTACGCCA**T**CAGCTCCAACCA | FAM |
| WT | 5´–GGCCTGCTGAAAATGACTGA–3´  3´–AGCAGCGTTACCTCTATCGT–5´ | TGCCTACGCCACCAGCTCCAACCA | HEX |

B

| ddPCR | Temperature (ºC) | Time |  |
| --- | --- | --- | --- |
| Step 1 | 95 ºC | 10 min |  |
|  | Ramp 2 ºC/s |  |  |
| Step 2 | 94ºC | 30 s | **Step 4**  Go to 2 x 40 cycles |
|  | Ramp 2 ºC/s |  |  |
| Step 3 | 64,5 ºC | 1 min |  |
|  | Ramp 2 ºC/s |  |  |
| Step 5 | 98 ºC | 10 min |  |
|  | Ramp 2 ºC/s |  |  |
| Step 6 | 4 ºC | ∞ |  |
|  | Ramp 2 ºC/s |  |  |

**Supplementary Table S6.** **(A)** qRT–PCR primers for mouse EMT genes. **(B)** qRT-PCR thermal cycling conditions for mouse EMT genes.

A

| Genes | Forward / Reverse | Gene Bank |
| --- | --- | --- |
| mVimentin | 5´–CGGCTGCGAGAGAAATTGC–3´  3´–CCACTTTCCGTTCAAGGTCAAG–5´ | NM_011701.4 |
| mE–Cadherin | 5´–GGTTTTCTACAGCATCACCG–3´  3´–GCTTCCCCATTTGATGACAC–5´ | NM_009864.3 |
| mN–Cadherin | 5´–TGAAGATGGCCGTTGGAGGCTG–3´  3´–ACTGGGTCATCCCGCCAATCA–5´ | NM_007664.5 |
| mTwist | 5´–CCGGAGACCTAGATGTCATTGT–3´  3´–CCACGCCCTGATTCTTGTGA–5´ | NM_011658.2 |
| mSnail | 5´–TCCAAACCCACTCGGATGTGAAGA–3´  3´–TTGGTGCTTGTGGAGCAAGGACAT–5´ | NM_011427.3 |
| mGADPH | 5′–CATGGCCTTCCGTGTTCCTA–3′  5′–GCGGCACGTCAGATCCA–3′ | NM_001289726.1 |

*m: mouse

B

| qRT-PCR  stages | Temperature  (ºC) | Time |  |
| --- | --- | --- | --- |
| Holding stage | 50 ºC | 2 min |  |
|  | 95 ºC | 10 min |  |
| Cycling stage | 95 ºC | 15 s | 40 cycles |
|  | 60 ºC | 1 min |  |
| Melt Curve stage | 95 ºC | 15 s |  |
|  | 60 ºC | 1 min |  |
|  | 95ºC | 30s |  |
|  | 60 ºC | 15 s |  |
